# Concordance between GPT-4 and a multidisciplinary tumor board in pancreatic cancer: A prospective pilot study

**DOI:** 10.1007/s00423-026-04242-9

**Published:** 2026-09-17

**Authors:** Fiete Gehrisch, Kürsat Kirkgöz, Antonie Willner, Faik G. Uzunoglu, Marianne Sinn, Jan Bardenhagen, Mara R. Goetz, Andreas Brandl, Felix Nickel, Anna Nießen, Thilo Hackert, Thilo Welsch

**Affiliations:** 1 Department of General, Visceral, Transplant and Thoracic Surgery, University Medical Center Hamburg-Eppendorf, Hamburg, Germany; 2 Center for Oncology, II. Medical Clinic and Polyclinic, University Medical Center Hamburg-Eppendorf, Hamburg, Germany; 3 Department of General, Visceral and Tumor Surgery, Krankenhaus Nordwest, Frankfurt, Germany

**Keywords:** Artificial intelligence, Generative AI, ChatGPT, Pancreatic ductal adenocarcinoma, Clinical decision making

## Abstract

**Background:**

Large language models (LLMs) such as GPT-4 are being evaluated for their use as supportive tools in oncological treatment planning. However, in pancreatic cancer, current studies are confined to predefined question–answer formats, while studies specifically investigating real-world scenarios that benchmark LLM performance against multidisciplinary tumor board (MDT) decisions are lacking.

**Methods:**

This prospective comparative analysis evaluated treatment and diagnostic recommendations for patients with newly diagnosed or suspected pancreatic cancer between an MDT and GPT-4. Using MDT referrals, clinical data were entered into a clinical data matrix and submitted to GPT-4 for therapeutic and diagnostic recommendations. Outputs were assessed before and after additional prompting with 41 high-ranking abstracts relevant to pancreatic cancer care. The primary endpoint was the concordance of recommendations between the MDT and GPT-4 before and after literature-based prompting.

**Results:**

Between September 1, 2024 and March 31, 2025, 45 patients were enrolled. The overall concordance rate between the MDT and GPT-4 was 73.3% (κ = 0.64, *p* < 0.0001) and did not improve following literature prompting. Discordance most often occurred in complex clinical scenarios. Concordance was highest in cases of metastatic disease (90.0%) and in neoadjuvant settings (90.0%) while it was lowest in patients requiring additional diagnostic workup (50.0%).

**Conclusions:**

GPT-4 demonstrated substantial agreement with MDT recommendations in patients with newly diagnosed or suspected pancreatic cancer. However, specific abstract prompting did not enhance the rate of concordance and GPT-4’s limitations in individualized or complex contexts underscore the need for a cautious future integration into oncologic workflows.

**Supplementary Information:**

The online version contains supplementary material available at https://doi.org/10.1007/s00423-026-04242-9.

## Introduction

Pancreatic cancer is a leading cause of cancer mortality worldwide, accounting for approximately 140,000 deaths annually in Europe alone [1]. The clinical management of pancreatic cancer is centralized and requires individual, interdisciplinary expert recommendations. As protocols and guidelines for pancreatic cancer continue to evolve, patient outcomes depend on appropriate therapeutic choices, while oncological decision-making remains a highly complex task [2]. As such, artificial intelligence (AI)-based large language models (LLMs) – commonly referred to as chatbots – like ChatGPT may serve as supportive tools to aid clinicians in navigating the complexities of pancreatic cancer care, potentially improving patient outcomes [3].

Since its launch in November 2022, OpenAI’s Chat Generative Pre-trained Transformer (ChatGPT) has become one of the most widely adopted platforms, demonstrating superior capabilities compared to its predecessors and competitors [4, 5]. However, most LLMs are trained predominantly on non-medical datasets [6].

One of the most frequently evaluated applications is ChatGPT’s ability to assist in clinical decision-making [7, 8]. Regarding the use of ChatGPT for oncological treatment planning, previous studies in a variety of different cancers, including gastrointestinal malignancies, have reported variable but promising rates of concordance between ChatGPT’s suggestions and those of medical professionals [9, 10]. In pancreatic cancer, current studies comprise predefined question–answer formats, while studies specifically investigating real-world clinical scenarios and applications are lacking [3, 11–13]. However, to ensure reliability and safety of AI-generated outputs, rigorous benchmarking against standard of care protocols remains imperative before LLMs can be integrated into clinical workflows [14, 15].

The present study aims to evaluate GPT-4’s ability to generate treatment recommendations for patients with pancreatic cancer by benchmarking its outputs against expert decisions from a university multidisciplinary tumor board (MDT). Using clinical patient data derived from tumor board referrals and standardized prompts, we assessed the concordance between GPT-4–generated diagnostic and therapeutic recommendations and those made by the MDT. Furthermore, we evaluated whether additional prompting with currently relevant literature in pancreatic cancer care could improve the clinical relevance and concordance of GPT-4’s outputs.

## Material and methods

### Study design and participants

This prospective comparative study evaluated treatment and diagnostic recommendations for patients with pancreatic cancer from an MDT to those of GPT-4 (OpenAI, San Francisco, United States) [16]. Prior to study initiation, the study was reviewed and approved by the local ethics committee of the University Medical Center Hamburg-Eppendorf (Ethics Commission Hamburg) (#2024–101273-BO-ff). All consecutive patients with newly suspected or histologically proven pancreatic ductal adenocarcinoma (PDAC) discussed in the MDT of the University Medical Center Hamburg-Eppendorf between September 2024 and March 2025 were prospectively assessed for enrollment. Patients were aged 18 years or older. Patients who were not newly diagnosed, or who had secondary malignancies or pancreatic malignancies other than ductal adenocarcinoma, were excluded. Before inclusion all patients provided written informed consent.

### Multidisciplinary tumor board

According to local routine, cases were referred to the MDT from different outpatient clinics using a standardized tumor board referral. During each MDT meeting, at least one senior representative from the Department of Radiology, the Department of General, Visceral and Thoracic Surgery, the Department of Oncology, the Department of Radiation Oncology, the Department of Pathology, and from the Department of Gastroenterology were present. Resectability was assessed as proposed by Isaji et al*.* [17] and all recommendations regarding treatment or diagnostic evaluation were made in line with the German Pancreatic Cancer guidelines [18] as well as NCCN [19] and ESMO [20] guidelines.

### Systematic literature research

To identify high-impact studies relevant to current pancreatic cancer care, we conducted a systematic literature search of the PubMed database (NLM/NCBI) (Supplementary Fig. 1). The following search strategy was used: pancreatic cancer AND chemotherapy OR radiation OR irreversible electroporation OR chemoradiation OR immunotherapy OR resection AND adjuvant OR neoadjuvant OR palliative AND survival OR progression-free OR disease-free AND “N Engl J Med” [journal] OR “Lancet” [journal] OR “Lancet Oncol” [journal] OR “J Clin Oncol” [journal] OR “JAMA” [journal] OR “JAMA Surg” [journal] OR “Ann Surg” [journal] AND randomized OR randomized-controlled OR RCT.

### Data collection

Patients’ clinical data were anonymized and, following the MDT meeting, transferred from the MDT referral to a standardized clinical data matrix containing demographic information, clinical characteristics, diagnostic data, radiological and endoscopic findings, resectability status, and laboratory findings (Supplementary Table 1). The transferred data consisted exclusively of information documented in the MDT referral and were therefore available to all board members. Exceptions were made for CA19-9 as well as CEA levels, and radiologic resectability criteria, that were if unavailable, retrieved from the electronic patient file. Data on radiological imaging and other diagnostic procedures was entered using full reports.

### Model and prompting strategy

All prompting was performed by the same investigator between September 1, 2024, and March 31, 2025, using GPT-4 [16] through the ChatGPT web interface (OpenAI, San Francisco) using an individual monthly subscription of ChatGPT Plus. Interactions were conducted under default model behavior, and no adjustments were made to sampling parameters. No separate system prompts were used. Memory and web access were enabled and a fresh conversation was used for each case. Chatbot prompts were provided in English and clinical data were provided in German. The standardized clinical data matrix contained an informational paragraph describing its purpose and the surgical experience available at the treating center (Supplementary Table 1). Within the same session, the matrix was uploaded along with a prompt to generate a treatment recommendation for the case at hand and to reconsider the initial recommendation after additional literature prompting (“*This is clinical data from a multidisciplinary tumor board referral of a patient with proven or suspected pancreatic cancer. Generate a recommendation for the multidisciplinary tumor board. After an initial recommendation you will receive 41 abstracts of studies currently relevant in pancreatic cancer care. Considering these studies, again provide a recommendation for the multidisciplinary tumor board.”)* The first response generated by GPT-4 following the initial prompt and after additional prompting with literature was recorded and used for analysis. No retry logic was implemented and no iterative refinement prompting was used. No re-asking or rephrasing was performed if the model produced an incomplete or unexpected response. If a response was truncated due to technical interface limitations, the continuation function was used without altering the prompt.

### Outcomes

The primary outcomes of this study were, first, the rate of concordance between treatment and diagnostic recommendations generated by GPT-4 and those of the MDT, and second, to examine whether additional literature prompting could improve this concordance. A treatment strategy (resection/exploration, neoadjuvant therapy, systemic palliative treatment, or recommendation for additional diagnostic workup) was assigned to each MDT and GPT-4 recommendation by a single blinded reviewer. Treatment recommendations by GPT-4 were classified both before and after literature prompting. All data were reviewed by a senior surgical oncologist. Concordance was defined as an agreement between GPT-4 and the MDT on the overall treatment strategy. Additional recommendations generated by GPT-4, surpassing a general treatment strategy, were further assessed for their clinical relevance and medical accuracy in the context of each individual clinical case.

### Statistical analysis

Data are reported as n (%) or mean (SD) for laboratory findings. Tumor markers and age are reported as median (IQR). Categorical variables were compared using the Chi-square test or Fisher’s exact test, as appropriate. Continuous variables were compared using two-tailed Welch’s t-test or the Wilcoxon test. Concordance between ChatGPT’s treatment recommendations and those of the MDT was assessed using Cohen’s Kappa coefficient (κ), with the MDT decision considered the reference standard. The strength of agreement was interpreted according to the Landis and Koch classification. Statistical significance of the observed agreement was assessed using the asymptotic test for κ. Statistical significance was set at *p* < 0.05. All statistical analyses were performed using JMP® version 18.2.1 (SAS Institute Inc., Cary, USA).

## Results

### Patient characteristics

Between September 1, 2024, and March 31, 2025, 74 patients were screened for eligibility, and 45 patients were enrolled (Supplementary Fig. 2). Patient characteristics for the overall patient cohort and by matching status are summarized in Table 1. The median age at presentation was 67 (IQR 61–72) years, and 19 (42.2%) patients were female. At diagnosis 19, patients (50.0%) had T1-T2 stage tumors, 15 (39.5%) had T3-T4 stage tumors, 13 (34.3%) were node positive and 11 (28.9%) had signs of metastatic disease. The pancreatic head was the most common tumor location, occurring in 27 patients (69.2%). With respect to resectability, 15 patients (34.1%) were classified as primary resectable, 12 (27.3%) as borderline resectable, six (13.6%) as locally advanced, and 11 (25.0%) as unresectable metastatic.

**Table 1:** Patient characteristics

Patient cohort *N* = 45	
Sex	
Female	19 (42.2)
Male	26 (57.8)
Age, years	67 (61–72)
ECOG	
0	37 (82.2)
1	8 (17.8)
cTNM	
T1-2	19 (50.0)
T3-4	15 (39.5)
TX	4 (10.5)
N0	23 (60.5)
N +	13 (34.2)
NX	2 (5.3)
M0	25 (65.7)
M +	11 (28.9)
MX	2 (5.3)
Tumor location	
Head	27 (69.2)
Body	9 (23.1)
Tail	3 (7.7)
Cumulative resectability	
Resectable	15 (34.1)
Borderline	12 (27.3)
Unresectable (LA)	6 (13.6)
Unresectable (M)	11 (25.0)
Tumor markers	
CA19-9, kU/l	188 (35–2329)
CEA, μg/l	2 (1–4)
Smoking	
yes	4 (100.0)
no	0 (0.0)
Laboratory findings	
Hemoglobin, g/dl	13.4 (1.7)
Creatinine, mg/dl	0.9 (0.2)
Bilirubin, mg/dl	4.2 (5.5)
Therapeutic intent	
Palliative	8 (21.1)
Curative	30 (78.9)

Data are given as *n* (%) or mean (SD) for laboratory findings. Tumor markers and age are reported as median (IQR). Because of cases with missing data, the numbers in the different categories do not always add up to the total number of cases. Data categories with fewer than two observations were excluded. *ECOG* Eastern Cooperative Oncology Group Performance Status. *cTNM* Clinical Tumor–Node–Metastasis classification; *T* Tumor stage; *N* Lymph node status; *M* Metastatic spread; *X* Assessment not possible; *LA* Locally advanced. *p < 0.05

### Concordance between MDT and GPT-4

**Fig. 1:**
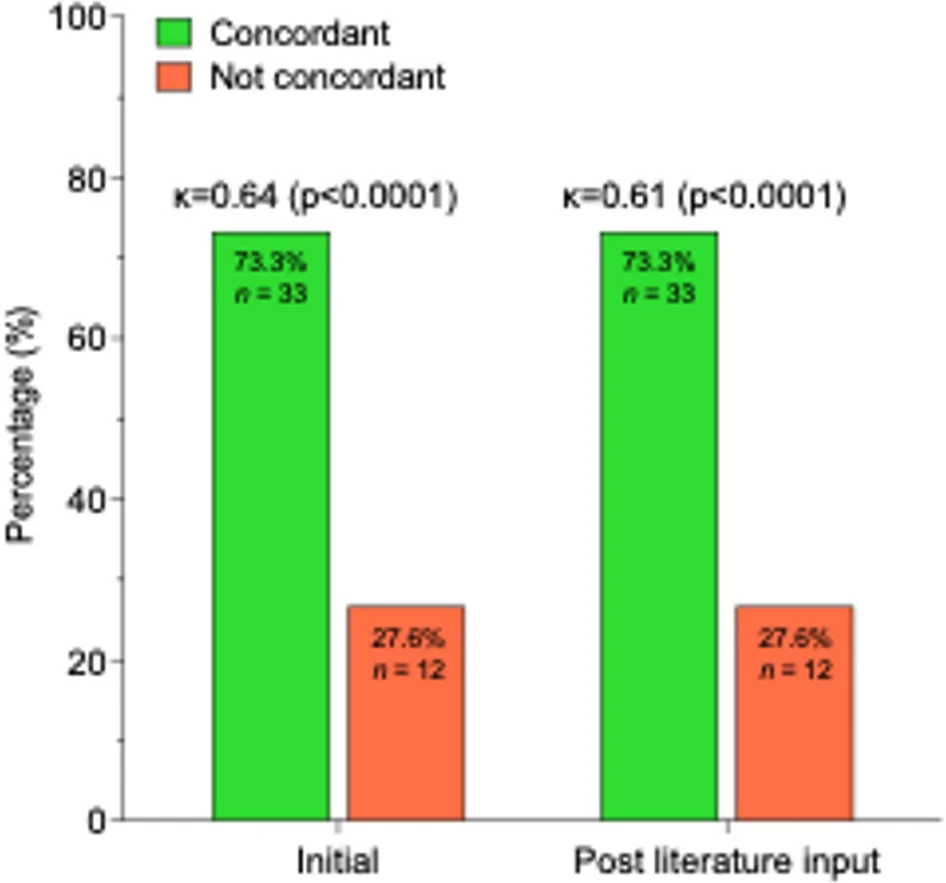
Concordance between GPT-4 and multidisciplinary tumor board decision. Bar graph illustrating the concordance between GPT-4 and multidisciplinary tumor board (MDT) recommendation before and after a structured literature input. Matching was defined as agreement regarding a recommendation for resection/exploration, neoadjuvant treatment, palliative systemic treatment or additional diagnostic work up

Concordance between MDT and GPT-4 recommendations was evaluated both before and after additional prompting with 41 high-ranking abstracts. The observed agreement before literature-based prompting was 73.3% (33 cases, κ = 0.64, *p* < 0.0001, Fig. 1). Additional literature prompting did not result in any cases in which GPT-4’s recommendations became concordant with those of the MDT (33 cases, 73.3%, κ = 0.61, *p* < 0.0001, Fig. 1), indicating substantial agreement in both scenarios.

Significant differences in baseline characteristics between both groups (MDT and GPT-4 concordance = matched group vs. not matched group) were only observed regarding M-status. In the matched group 10 out of 26 patients (38.5%) had metastatic disease compared to 1 out of 12 patients (8.3%) in the not matched group (*p* = 0.0408, Table 2). Among the 12 non-concordant cases, the presumed cause of discordance was an inadequate capture of clinical complexity by GPT-4 in three cases (25.0%), incomplete data in the MDT referral in three cases (25.0%) and a disregard of essential clinical data by GPT-4 in one case (8.3%). Regarding the remaining five non-matching cases, no evident cause of discordance could be identified. A detailed case-level analysis of all non-concordant recommendations is provided in Table 3.

**Table 2:** Patient characteristics by matching status

	*N*	Matched	Not Matched	*p-Value*
	45	33 (73.3)	12 (26.7)	
Sex				0.9637
Female	19	14 (42.4)	5 (41.7)	
Male	26	19 (57.6)	7 (58.3)	
Age, years	45	66 (61–70)	71 (62–75)	0.1610
ECOG				0.9059
0	37	27 (81.8)	10 (83.3)	
1	8	6 (18.2)	2 (16.7)	
cTNM				
				0.9490
T1-2	19	13 (50.0)	6 (50.0)	
T3-4	15	10 (38.5)	5 (41.7)	
TX	4	3 (11.5)	1 (8.3)	
N0	23	13 (50.0)	10 (83.3)	0.0933
N +	13	11 (42.3)	2 (16.7)	
NX	2	2 (7.7)	0 (0.0)	
M0	25	14 (53.8)	11 (91.7)	***0.0408***
M +	11	10 (38.5)	1 (8.3)	
MX	2	2 (7.7)	0 (0.0)	
Tumor location				0.8907
Head	27	19 (67.9)	8 (72.7)	
Body	9	7 (25.0)	2 (18.1)	
Tail	3	2 (7.1)	1 (9.1)	
Cumulative resectability				0.4805
Resectable	15	11 (33.3)	4 (36.4)	
Borderline	12	8 (24.2)	4 (36.4)	
Unresectable (LA)	6	4 (12.1)	2 (18.2)	
Unresectable (M)	11	10 (30.3)	1 (9.1)	
Tumor markers				
CA19-9, kU/l	43	188 (41–3781)	120 (26–1913)	0.4984
CEA, μg/l	38	2 (1–4)	3 (1–4)	0.8466
Smoking				1.0
yes	4	4 (100.0)	0 (0.0)	
no	0	0 (0.0)	0 (0.0)	
Laboratory findings				
Hemoglobin, g/dl	26	13.2 (1.5)	13.9 (2.3)	0.4595
Creatinine, mg/dl	26	0.9 (0.2)	0.8 (0.1)	0.3062
Bilirubin, mg/dl	31	3.6 (4.9)	5.8 (6.8)	0.3794
Therapeutic intent				0.2897
Palliative	8	7 (25.0)	1 (10.0)	
Curative	30	21 (75.0)	9 (90.0)	

Data are given as *n* (%) or mean (SD) for laboratory findings. Tumor markers and age are reported as median (IQR). Because of cases with missing data, the numbers in the different categories do not always add up to the total number of cases. Data categories with fewer than two observations were excluded. *ECOG* Eastern Cooperative Oncology Group Performance Status; *cTNM* Clinical Tumor–Node–Metastasis classification; *T* Tumor stage; *N* Lymph node status; *M* Metastatic spread; *X* Assessment not possible; *LA* Locally advanced. *p < 0.05

**Table 3:** Case-level analysis of 12 non-concordant recommendations

Case	MDT	GPT-4	Presumed cause for discordance	Fraction of discordant cases (%)	Context
1	R/E	NA / DIAG**$**	*Inadequate capture of clinical complexity*	25.0%	Anatomically resectable but biologically borderline tumor without prior histologic confirmation. Markedly elevated CA19-9 (3131.6 kU/L), discordant with CT findings. MDT to recommend laparoscopic exploration to exclude peritoneal metastasis and assess resectability. GPT-4 initially recommended NA and after literature prompting changed its reccomendation to histological confirmation via FNA
2	NA	R/E			Extracapsular tumor extension in a laparoscopically retrieved lymph node. MDT recommended NA despite anatomic resectability. GPT-4 advised primary resection
3	R/E	NA			Borderline resectable patient with endoscopically untreatable peptic stenosis and malnutrition. MDT recommended primary resection. GPT-4 recommende neoadjuvant therapy based on borderline resectability
4	NA	DIAG	*Incomplete referral data*	25.0%	Patient with three unsuccessful biopsy attempts (not noted on the MDT referral) and borderline disease. MDT recommended NA, while GPT-4 recommended histological confirmation via EUS
5	NA	R/E			Anatomically resectable patient with CA19-9 > 500 kU/L recorded on referral. GPT-4 recommended NA. Patient file revealed that before the MDT conference, following biliary drainage, CA 19–9 levels had normalized, and the initial CA 19–9 elevation was attributed to cholestasis. MDT recommended primary resection
6	DIAG	PAL			Anatomically resectable patient with prior histological confirmation. CT scan with, indeterminate pulmonary nodule but no radiologoical evidence highly suggestive of distant metastasis. Incorrectly, the MDT referral documented an M1 status. MDT recommended exclusion of pulmonary metastasis via VATS. GPT-4 recommended PAL
7	DIAG	R/E	*Disregard of essential clinical data*	8.3%	Although anatomic resectability was suspected, due to technical issues resulting in reduced image quality, CT report explicitly stated that anatomic resectability could not be fully determined. MDT recommended a repeat scan to clarify resectability. GPT-4 advised to proceed with primary resection
8	R/E	DIAG	*No evident cause of discordance*	41.7%	-
9	R/E	DIAG			-
10	R/E	DIAG			-
11	DIAG	NA			-
12	R/E	NA			-

Abbreviations: *R/E* Resection/exploration; *NA* Neoadjuvant treatment; *PAL* Palliative systemic treatment; *DIAG* Additional diagnostic workup. $: Only case, among all 45 evaluated cases, with a change of recommendation by GPT-4 after literature prompting. *VATS* Video-assisted thoracoscopic surgery

### Data availability

**Fig. 2:**
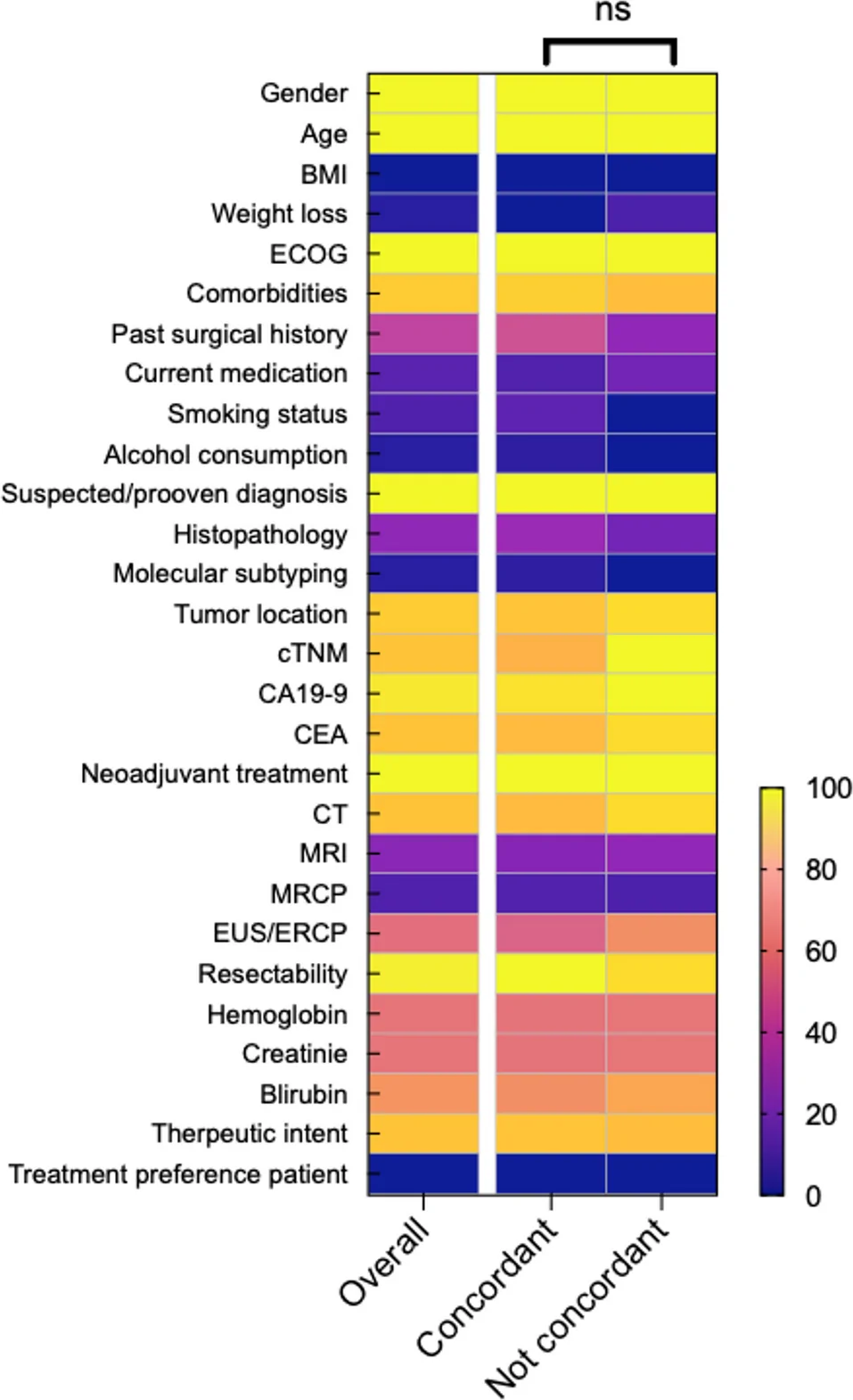
Availability of clinical data. Heatmap illustrating the presence or absence of clinical data within the patient matrix across all patients and by concordance status. Yellow indicates missing data; blue indicates available information. The bracket indicates that univariate analysis revealed no significant differences in data availability between both groups. ns = not significant

To simulate a real-world clinical scenario, data were collected exclusively using the MDT referral. Missing variables were considered inherent to the study design and retained throughout the subsequent statistical evaluation. The presence or absence of clinical data within the matrix, in the overall patient cohort and by concordance status, is illustrated in a heatmap (Fig. 2). Data on age, gender, ECOG status, suspected or proven diagnosis and neoadjuvant therapy were available in all 45 patients, while data on weight loss and alcohol consumption were only available in one patient (2.2%). Data regarding patients’ treatment preference were absent in all cases. Univariate analysis revealed no significant differences in data availability between the matched and not matched group (Fig. 2).

### Recommendations and abstract referencing

**Fig. 3:**
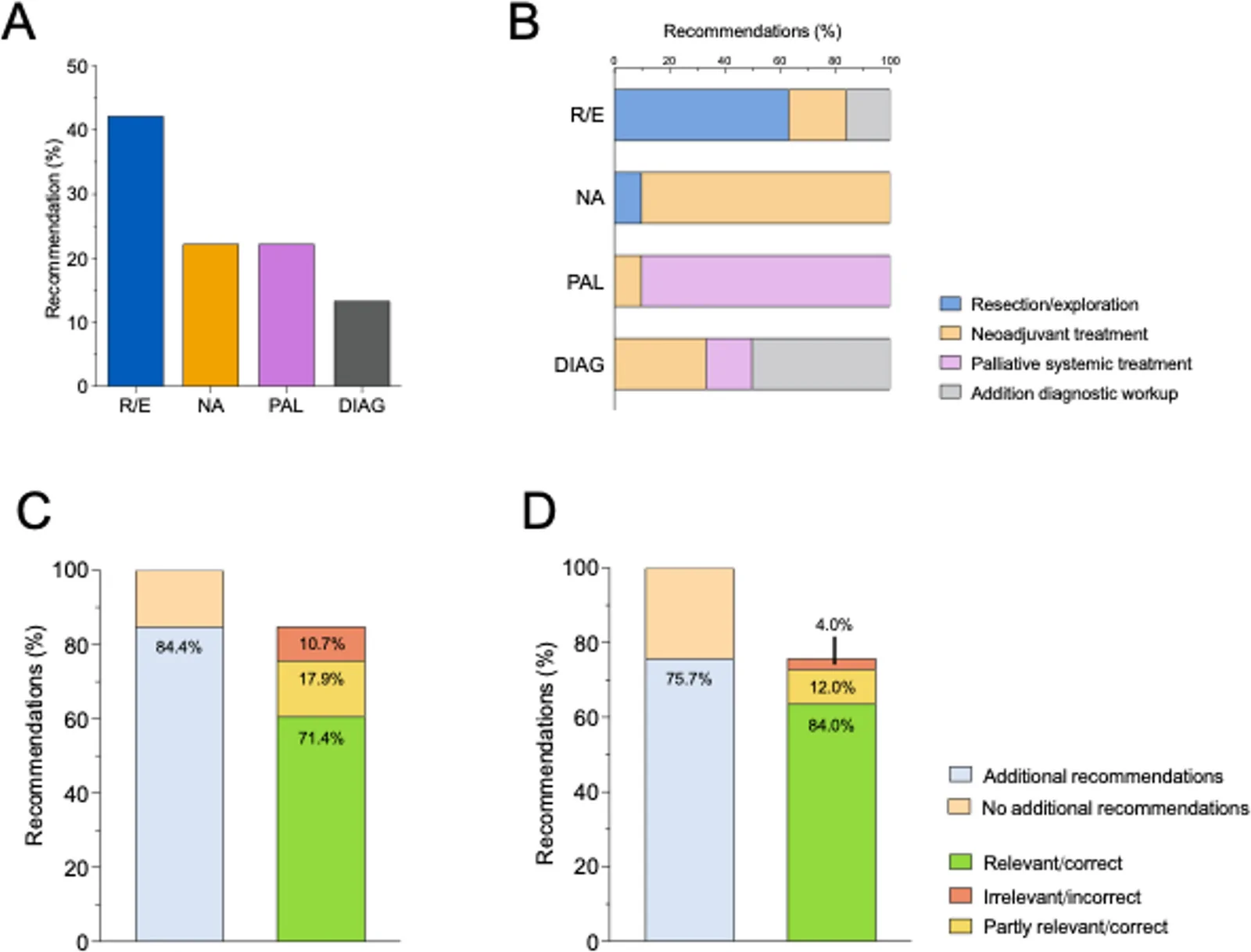
Recommendations by the multidisciplinary tumor board and GPT-4. Distribution of treatment recommended by the multidisciplinary tumor board (MDT), expressed as percentages across the categories resection/exploration, neoadjuvant treatment, palliative systemic treatment, and additional diagnostic workup (**A**). GPT-4’s recommendations in relation to the MDT as reference standard (**B**). Rates and clinical relevance of additionally generated recommendations by GPT-4 in concordant cases before (**C**) and after (**D**) additional prompting with 41 abstracts. R/E = resection/exploration. NA = neoadjuvant treatment. PAL = palliative systemic treatment. DIAG = additional diagnostic workup

The MDT recommended a resection/exploration in 19 cases (42.2%), neoadjuvant therapy and systemic palliative treatment in 10 cases each (22.2%) and an additional diagnostic workup in 6 cases (13.3%) (Fig. 3A). The rate of discordance was highest when the MDT recommended additional diagnostic workup (50.0%), followed by exploration/resection (36.8%), and was lowest when neoadjuvant (10.0%) or palliative systemic treatment (10.0%) was advised by the MDT (Fig. 3B).

Among concordant cases, GPT-4 provided additional recommendations beyond treatment strategy in 28 of 33 cases (84.8%) (Fig. 3C). These recommendations were retrospectively reevaluated by clinical experts. In these 28 cases, the additional recommendations were rated clinically relevant and accurate in 20 cases (71.4%), partially relevant in 5 (17.9%), and irrelevant in 3 cases (10.7%) (Fig. 3C). Similarly, new recommendations generated after literature-based prompting were evaluated. Additional literature-based prompting generated new recommendations in 25 out of 33 concordant cases (75.7%), of which 21 (84.0%) were assessed as relevant and accurate, 3 (12.0%) as partially relevant/incorrect and 1 (4.0%) as irrelevant and incorrect (Fig. 3D).

Supplementary Table 2 summarizes how often each of the 41 abstracts was referenced by GPT-4 across all recommendations. Notably, 31 abstracts (75.4%) were not cited at all. Among the 10 cited abstracts the three most frequently referenced, each cited nine times, were all published in 2024.

## Discussion

LLMs, such as OpenAI’s ChatGPT, are emerging as a transformative force in various sectors, offering potential applications in medicine, including oncology decision-making [21]. However, as their role in healthcare expands and concerns regarding their reliability grow, rigorous validation of their accuracy remains imperative to ensure a safe future integration into clinical workflows [3, 22].

A key finding of our study is that, using real-world clinical data from patients with newly diagnosed or suspected PDAC, GPT-4 achieved substantial agreement with an MDT on treatment recommendations (Fig. 1). Similarly, other studies have reported variable but encouraging rates when benchmarking GPT-4’s performance against clinical experts in oncological decision-making settings. Reported concordance rates for example include 72.5% in colorectal cancer [9], 62.1% in renal cell carcinoma [23] and 67.5% across malignancies of various origins [10]. In patients with pancreatic cancer, Aghamaliyev et al*.* reported an alignment of overall treatment strategy between GPT-3.5 and an MDT of 84.0% across 39 PDAC cases, but their study design lacked a standardized prompting protocol, limiting the interpretability of their finding [13]. Other studies assessing the role for ChatGPT in PDAC related decision-making have relied on predefined clinical questions rather than real-world data but have reported similar rates of alignment with guidelines and expert opinions, ranging from 60.0% [11, 24] to 78.6% [3]. However, other studies have reported rates of accuracy and concordance far below ours, which may be attributed to heterogeneity in study design and methodology, including the use of earlier ChatGPT versions as well as different prompting strategies and concordance measurements [25–27].

A notable observation in this study was that metastatic disease was more frequent in cases rated concordant (*p* = 0.0408, Table 2) and that the rate of concordance was highest when the MDT recommended systemic palliative treatment (Fig. 3). Arguably, this difference may stem from the fact that advanced metastatic disease is typically managed with standardized systemic therapy, whereas resectable or borderline cases demand a more complex, individualized treatment strategies [18–20]. This also underlines the role of the specialized radiologists and surgeons in assessing potential resectability in advanced cases. Along the same lines, non-concordant cases were attributable, at least in part, to particularly complex clinical scenarios (Table 3). While these observations remain correlative, they are consistent with the notion that LLMs, in their current form, are not capable of serving as stand-alone decision-makers and that expert judgment remains a critical factor [25].

Another finding was that agreement did not improve after prompting with 41 abstracts (Fig. 1). Although GPT-4 cited only 10 (24.4%) of these abstracts, the cited publications were predominantly recent (Supplementary Table 2). The limited ability of LLMs such as GPT-4 to adequately contextualize or individualize treatment recommendations has been reported previously and reflects inherent limitations in their architecture and retrieval mechanisms [28]. Rather than performing a deliberate, evidence-based evaluation of the provided data, GPT-4 generates outputs through probabilistic pattern recognition, with a tendency to favor more recent data or language patterns [29, 30]. A related concern is the variability of outputs across clinically comparable cases, which was also observed in this study [31]. In some instances, responses were brief and lacked contextual detail, whereas in other seemingly similar cases, they were substantially more comprehensive (Fig. 3C/D). Such variability may arise from the existence of multiple plausible outputs in LLMs, where sensitivity to contextual variations can shift response trajectories and stochastic sampling may favor brevity over elaboration [32]. Interestingly, although additional prompting did not enhance concordance, the additional information generated was assessed as relevant and accurate in 84.0% (Fig. 3D). This suggests that GPT-4 can integrate the provided information into its responses; however, this capability does not necessarily translate into evidence-based reasoning or improved clinical decision support.

The case of a patient for whom GPT-4 recommended a resection, while the CT report explicitly stated that resectability could not be determined due to technical issues, highlights a recognizable risk associated with integrating LLMs in high-stakes domains like healthcare. Observations like these have also been made by other authors and likely reflect poorly calibrated and unreliable confidence estimates as well as the model’s tendency to produce hallucinations [3, 10, 26, 33, 34]. In this study GPT-4 was used in its default form without prior prompting or fine-tuning tailored to pancreatic cancer care. However, the accuracy of general-purpose LLMs can be substantially improved through model fine-tuning or retrieval-augmented generation (RAG) [35]. Notably, Ke et al. demonstrated that a GPT-4–based RAG model incorporating international clinical guidelines achieved accuracy rates above 95% across various surgical scenarios and outperformed human-generated responses [36]. In pancreatic oncology, such approaches could generate responses more precisely tailored to regional or institutional guidelines and better aligned with the nuanced demands of real-world patient care.

This study is subject to several limitations. As this is a single-center pilot study, its relatively small sample size and lack of outcome data restrict the generalizability of our findings. GPT-4’s recommendations were compared with the decisions of an MDT from a German university hospital. Because these decisions were likely influenced by German pancreatic cancer guidelines [18], which in some aspects differ from other international guidelines, our findings may be limited in an international clinical context. No advanced prompt engineering or model fine-tuning was employed, potentially limiting the assessment of GPT-4’s full capabilities. Concordance was assessed by a single rater, introducing the possibility of bias. Although concordance was the primary outcome measure, the variability of model outputs necessitated a descriptive rather than purely quantitative analytical approach. Another limitation of this study is the asymmetry in information available the MDT and GPT-4. GPT-4 was prompted using the standardized data matrix, which was primarily constructed from data documented in the MDT referral and supplemented by selected variables retrieved from the electronic patient record. The MDT by contrast, had access to specialist radiological review, live multidisciplinary discussion and potentially additional contextual information that was not captured in the referral. Consequently, discordance between GPT-4 and the MDT may partly reflect differences in the information available to each rather than differences in clinical reasoning alone. Nevertheless, this design was intended to reflect a real-world scenario in which an LLM is provided with structured clinical information available from routine documentation rather than the full range of information accessible to an MDT. Lastly, agreement with a local MDT decision cannot be interpreted as evidence of accuracy, safety, improved patient care, or readiness for clinical implementation.

Taken together, our results demonstrate that GPT-4’s treatment recommendations largely align with guideline-based expert opinions in pancreatic cancer. Given the rapid advancements of LLMs, these findings support a potential role of LLMs in oncologic decision-making. However, important limitations remain, particularly with regard to reliability in real-world clinical settings. Safe integration of LLMs into clinical workflows will therefore require further refinements and rigorous oversight.

## Supplementary Information

Below is the link to the electronic supplementary material.

Supplementary Material 1 (DOCX 192 KB)

## Data Availability

All data generated or analyzed during this study are available from the corresponding author upon request.

## Declarations

### STROBE

This study is reported in accordance with the Strengthening the Reporting of Observational Studies in Epidemiology (STROBE) guidelines.

## Abbreviations

AI: Artificial intelligence
LLM: Large language model
ChatGPT: Chat Generative Pretrained Transformer
MDT: Multidisciplinary tumor board
PDAC: Pancreatic ductal adenocarcinoma
RAG: Retrieval-augmented generation
