# Supplementary material for: Concordance between GPT-4 and a multidisciplinary tumor board in pancreatic cancer: A prospective pilot study
Source: Langenbecks Arch Surg. 2026 Sep 17;411(1):238. doi: 10.1007/s00423-026-04242-9 (PMC13588817; doi:10.1007/s00423-026-04242-9)
Supplement: Supplementary file 1 [file 423_2026_4242_MOESM1_ESM.docx]

**Supplementary Data**

**Concordance Between GPT-4 and a Multidisciplinary Tumor Board in Pancreatic Cancer: A Prospective Pilot Study**

Fiete Gehrisch^1^*^#^, Kürsat Kirkgöz^1^*, Antonie Willner^1^, Faik G. Uzunoglu^1^, Marianne Sinn^2^, Jan Bardenhagen^1^, Mara R. Goetz^1^, Andreas Brandl^3^, Felix Nickel^1^, Anna Nießen^1^, Thilo Hackert^1^, Thilo Welsch^1, 3^

^1^ Department of General, Visceral and Thoracic Surgery, University Medical Center Hamburg-Eppendorf, Hamburg, Germany.

^2^ II. Department of Medicine, University Medical Center Hamburg-Eppendorf, Hamburg, Germany.

^3^ Department of General, Visceral and Tumor Surgery, Krankenhaus Nordwest Frankfurt, Germany.

*Contributed equally.

^#^Corresponding author: Dr. med. Fiete Gehrisch, Department of General, Visceral and Thoracic Surgery, University Medical Center Hamburg-Eppendorf, Hamburg, Germany, Tel. +49 152 228 399 24, E-mail: [f.gehrisch@uke.de](mailto:f.gehrisch@uke.de). ORCID: 0009-0004-4790-7440.

**Supplementary Table 1. Clinical data matrix.**

Prospectivley collected patient data from tumor board referrals of patients with newly diagnosed or suspected pancreatic cancer were transferred to a clinical data matrix. If data on ECOG performance status, tumor markers and resectability were not present, they were retrieved from the electronic patient file and added to the matrix.

**Supplementary Table 2. Results of systematic literature search and abstract referencing by GPT-4.**

PMID=PubMed-ID.

**Supplementary Figure 1. Systematic literature search using the PubMed database.**

A systematic literature search was conducted using the PubMed database to identify key studies that discuss the treatment of pancreatic cancer and are currently relevant in clinical practice. The search was restricted to articles published in high-ranking clinical journals. 82 search results were full text screened, and 41 abstracts were included in this study.

**Supplementary Figure 2. Trial profile.**

74 patients with PDAC that were discussed in the multidisciplinary tumor board during September 2024 and March 2025 were screened. 29 patients were excluded and 45 patients with the initial diagnosis of PDAC were included in this study.
